# The Clinical and Immune Characteristics of Patients with Hepatitis-Associated Aplastic Anemia in China

**DOI:** 10.1371/journal.pone.0098142

**Published:** 2014-05-20

**Authors:** Huaquan Wang, Meifeng Tu, Rong Fu, Yuhong Wu, Hong Liu, Limin Xing, Zonghong Shao

**Affiliations:** 1 Department of Hematology, General Hospital, Tianjin Medical University, Tianjin, People's Republic of China; 2 Key Laboratory of Carcinogenesis and Translational Research (Ministry of Education), Department of Lymphoma, Peking University School of Oncology, Beijing Cancer Hospital and Institute, Beijing, People's Republic of China; Duke University, United States of America

## Abstract

Hepatitis-associated aplastic anemia (HAAA) is a variant of severe aplastic anemia (SAA) in which bone marrow failure follows an acute attack of hepatitis. Its pathogenesis is poorly understood. We investigated the prevalence of HAAA among cases of newly diagnosed SAA presenting to our hospital between January 1998 and February 2013, and analyzed the clinical and immune characteristics of HAAA and non-hepatitis-associated SAA (non-HASAA) patients. The prevalence of HAAA among cases of SAA was 3.8% (36/949), and the majority of patients (33/36) were seronegative for a known hepatitis virus. Compared with non-HASAA patients, HAAA patients had a larger proportion of CD8+ T cells, a lower ratio of CD4+/CD8+ T cells, and a smaller proportion of CD4+CD25+ regulatory T cells. There was no significant difference in peripheral blood count, bone marrow cellularity, or the number of blood transfusions received between HAAA and non-HASAA patients. HAAA patients had a higher early infection rate and more infection-related mortality in the first 2 years after diagnosis than non-HASAA patients, and their 2-year survival rate was lower. The results demonstrate that HAAA patients have a more severe T cell imbalance and a poorer prognosis than non-HASAA patients.

## Introduction

Hepatitis-associated aplastic anemia (HAAA) is a variant of severe aplastic anemia (SAA) in which bone marrow failure follows an acute attack of hepatitis [1]. It most frequently affects young male children and is often fatal if untreated. The subtypes of hepatitis viruses causing HAAA and the pathogenesis of the disease are currently poorly understood. We investigated the prevalence of HAAA among cases of newly diagnosed SAA presenting to our hospital in the last 15 years, and compared the clinical features, immune status, treatment response and prognosis of patients with HAAA with those with non-hepatitis-associated SAA (non-HASAA).

## Methods

### Patients

All patients diagnosed with SAA (according to the criteria of the International Aplastic Anemia Study Group) at the General Hospital, Tianjin Medical University, Tianjin, China, between January 1998 and February 2013 were included in this study (n = 949) (Table 1). The Ethics Committee of Tianjin Medical University approved the study protocol. Informed written consent was obtained from all patients or guardians in accordance with the Declaration of Helsinki. SAA was defined as pancytopenia with at least two of the following abnormalities: an absolute neutrophil count of <0.5×10^9^/L, a platelet count of <20×10^9^/L, and a reticulocyte count of <20×10^9^/L, in association with a bone marrow cellularity of <30%. Very severe aplastic anemia (VSAA) was diagnosed in the cases SAA with the neutrophil count < 0.2×10^9^/L. Patients were excluded if they had congenital anaplastic anemia (AA) (diagnosed by familial history, Mitomycin test and genetic tests), clonal diseases (by flow cytometry or genetic tests) or other autoimmune diseases. Patients were screened for paroxysmal nocturnal hemoglobinuria either by flow cytometry using anti-CD55 and anti-CD59 antibodies or using the Ham test for red blood cell fragility. Bone marrow cytogenetic studies were performed in all patients. Patients who had experienced acute hepatitis less than 6 months prior to developing SAA were diagnosed with HAAA (n = 36) [2]. All other SAA patients were defined as non-HASAA cases (n = 913). A diagnosis of hepatitis was made when serum aminotransferase levels were at least three times the upper limit of the normal range (normal range for alanine aminotransferase (ALT), 5–40 U/L; normal range for aspartate aminotransferase (AST), 8–40 U/L). A complete response was defined as a normal or near-normal blood count within a year after the initiation of therapy (hemoglobin concentration, 100 g/L; neutrophil count, 1×10^9^/L; and platelet count, 100×10^9^/L).

**Table 1:** Patient characteristics.

	HAAA	Non-HASAA	P
Patient number	36	913	
Median age (range); years	18 (5–62)	25 (3–87)	0.02
Patient gender; male/female	31/5	574/339	0.004
Interval between diagnosis of anemia and treatment Median (range); days	16 (1–210)	47 (1–1826)	0.001
Interval between hepatitis and anemia Median (range); days	32 (5–171)	-	
Severity of disease			0.02
VSAA	21	351	
SAA	15	562	
Median neutrophil count (range); × 10^9^/L	0.12 (0–1.12)	0.15 (0–1.4)	0.29
Median platelet count (range); × 10^9^/L	9 (0–32)	11 (0–43)	0.71
Median reticulocyte count (range); × 10^9^/L	14 (0–48)	13 (0–52)	0.86
Median Peak ALT (range); IU/L	1267 (472–2427)	86 (17–162)	0.001
Median Peak AST (range); IU/L	951 (395–2964)	66 (15–96)	0.001
Median Peak TBIL(range); μmol/L	176 (51–427)	26 (5–73)	0.001

HAAA, hepatitis-associated aplastic anemia; Non-HASAA, non-hepatitis-associated severe aplastic anemia; vSAA, very severe aplastic anemia; SAA, severe aplastic anemia; ALT, alanine aminotransferase; AST, aspartate aminotransferase; TBIL, total bilirubin.

### Therapy

Five of the 36 patients with HAAA died from severe infection or intracranial hemorrhage before treatment. The remaining 31 received immunosuppressive therapy (IST): 15 cases received rabbit anti-thymocyte globulin (ATG, Genzyme Polyclonals S.A.S, France) or pig anti-lymphocyte globulin (ALG, Wuhan Institute of Biological Products, China) in combination with cyclosporin (CsA) and hematopoietic growth factors (HGFs), 16 cases received androgen in combination with CsA and HGFs (Table 2).

**Table 2:** IST therapy of SAA patients.

	Num. of patients	
Therapy	HAAA	Non-HASAA
Rabbit ATG+CsA+HGFs	12	157
Pig ALG+CsA+HGFs	3	34
CsA+Androgen+HGFs	16	542
Supportive care	5	180
total	36	913

The data collected from the patients' records included the peripheral blood count, bone marrow features, liver function test results, parameters of the immune status, number of infections and cause of death. The Ethics Committee of Tianjin Medical University approved the study protocol. Informed written consent was obtained from all patients in accordance with the Declaration of Helsinki.

### Flow cytometric analysis

Peripheral blood T-cell subsets were measured by flow cytometry at diagnosis, using the following directly labeled antibodies: anti-CD3-PerCP, anti-CD4-FITC, anti-CD8-PE and anti-CD25-PE (BD Biosciences, San Jose, CA, USA). Data acquisition and analysis were carried out on a FACSCalibur flow cytometer, with CellQuest software version 3.1 (BD Biosciences).

### Enzyme-linked immunosorbent assay

Serum interleukin 2 (IL-2) and tumor necrosis factor-α (TNF-α) levels were measured using the appropriate human enzyme-linked immunosorbent assay kit (Promega, Madison, WI, USA) at diagnosis.

### Statistical analysis

Data are presented as the mean±SEM. For normally distributed data, the *t* test was used to compare two independent groups. A paired *t* test was used to compare two groups of paired data. The chi-square test was used for 2×2 tables and the log-rank test for time-dependent variables. Kaplan-Meier curves were used to estimate survival. A P value of < 0.05 was considered to be statistically significant. Statistical analysis was performed using SPSS software version 11.5 (SPSS Inc., Chicago, IL, USA).

## Results

### The clinical characteristics of HAAA

#### Prevalence of HAAA

A total of 949 patients with SAA were diagnosed in the 15-year study period between January 1998 and February 2013, and HAAA accounted for 36 cases (3.8%; Table 1).

#### Virology and liver function in HAAA

All 36 patients with HAAA had experienced acute hepatitis less than 6 months prior to a diagnosis of SAA. The median interval between a diagnosis of acute hepatitis and of HAAA was 32 days (range, 5–171 days). All the patients were seronegative for hepatitis virus A, C, D and E. Two cases (5.6%) were positive for hepatitis B virus antigens HBsAg and HBeAg. One case (2.8%) was positive for both HBsAb and HBeAb antibodies, while in 18 cases (50%) only HBsAb was positive. Fifteen cases (41.2%) were negative for hepatitis B virus antigens and antibodies. There were no causative virus for cytomegalovirus (CMV), herpes simplex virus (HSV), parvovirus B19 (HPVB19), and AIDS-related viruses, were not detected in any of the HAAA patients.

**Figure 1:**
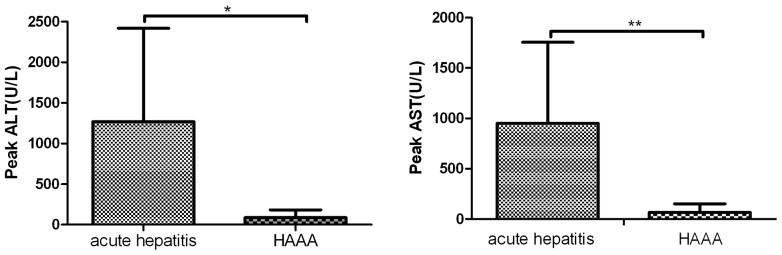
Levels of liver enzymes at diagnosis of acute hepatitis and hepatitis-associated severe aplastic anemia (HAAA). Alanine aminotransferase (ALT) and aspartate aminotransferase (AST) were at a lower level at diagnosis of HAAA than during the course of the preceding acute hepatitis. (*P<0.05, **P<0.05)

Serum ALT and AST measurements were used to assess liver function. Levels of both enzymes peaked during the course of the acute hepatitis, before the patient had been diagnosed with HAAA. The ALT level was significantly higher during the course of acute hepatitis (median, 1267±l150 U/L) than during the subsequent HAAA (median, 86±96 U/L; P<0.05). The AST level was also significantly higher during the course of acute hepatitis (median, 951±804 U/L) than during the course of HAAA (median, 66±86 U/L; P<0.05). In 33 (91.7%) of the 36 cases, either ALT, AST or both were at a lower level at the time of diagnosis of HAAA than they had been during the phase of acute hepatitis that preceded it. The serum level of ALT did not decline at the diagnosis of HAAA in three patients (8.3%; Fig. 1).

#### Features of the peripheral blood and bone marrow

There was no significant difference in the peripheral blood reticulocyte count or neutrophil count, in bone marrow cellularity (granulocyte/erythrocyte ratio and megakaryocyte count), or in the number of blood transfusions administered (including erythrocyte and platelet transfusions) between HAAA and non-HASAA patients.

### Immune status in HAAA

To gauge the immune status of the patients, we analyzed the proportion of peripheral blood T cells expressing CD4, CD8 and CD25. The proportion of CD4^+^ T cells in the peripheral blood was significantly lower (median, 20.5±12.8%) and the proportion of CD8^+^ T cells (median, 47.2±18.9%) significantly higher in HAAA patients than in non-HASAA patients (median, 34.3±14.8%; P<0.05, and median, 29.6±13.5%; P<0.05, respectively). The ratio of CD4^+^/CD8^+^ T cells in HAAA patients (median, 0.52±0.46) was significantly lower than that of non-HASAA patients (median, 1.26±0.72; P<0.05). The proportion of CD4^+^CD25^+^ T regulatory cells (Tregs) (median, 1.7±0.8%) was significantly lower than that of non-HASAA patients (4.2±2.1%; P<0.05).

**Figure 2:**
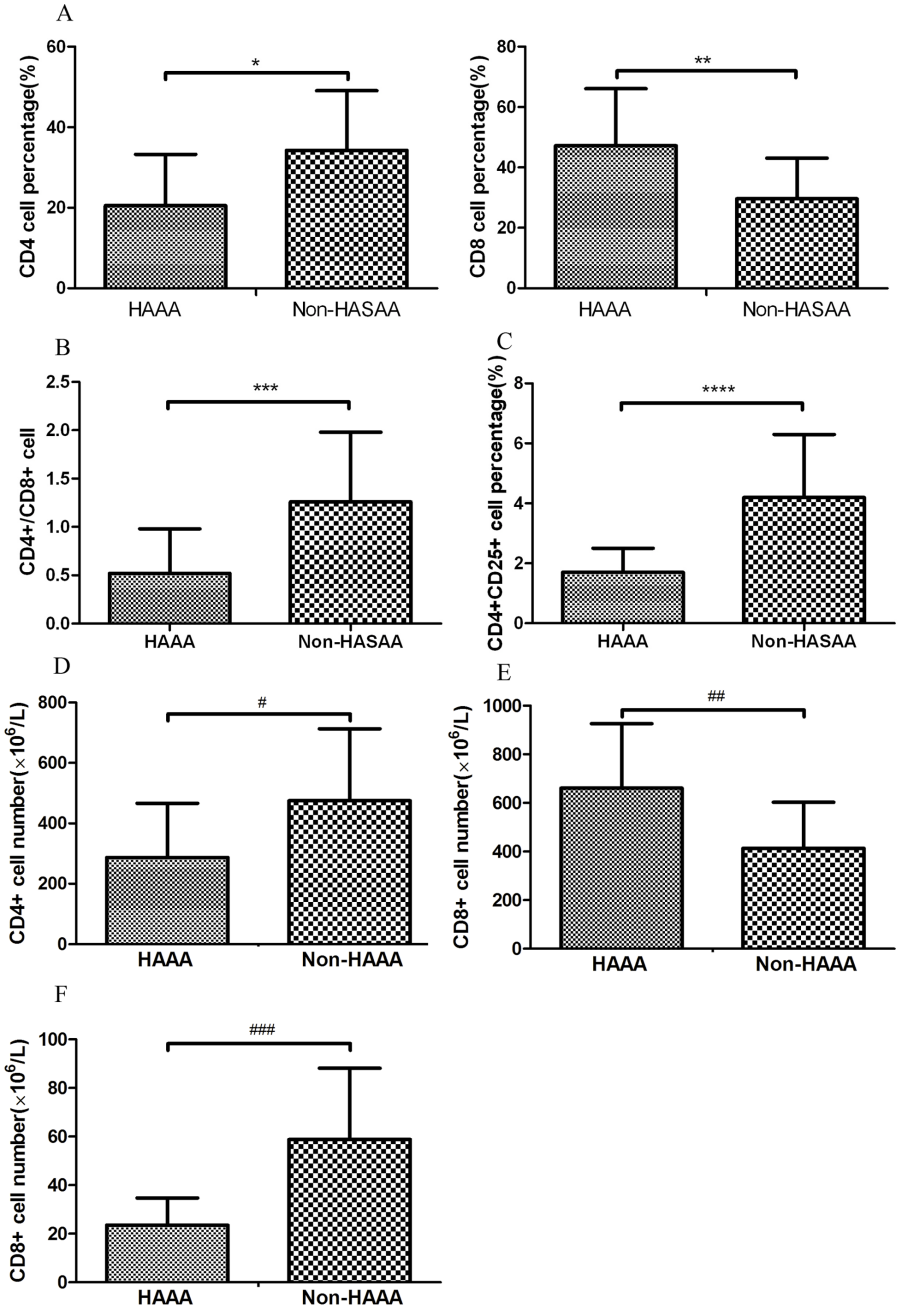
Analysis of T-cell subsets in peripheral blood of patients with hepatitis-associated aplastic anemia (HAAA) and non-hepatitis-associated severe aplastic anemia (Non-HASAA). We used a case-control study and collected random cases in non-HASAA patients as contrast group (ratio 1:4). (A) Compared with non-HASAA patients, those with HAAA had a smaller proportion of CD4+ T cells and a larger proportion of CD8+ T cells (*P<0.05, **P<0.05). (HAAA n = 36, Non-HASAA n =  144) (B) The ratio of CD4+/CD8+ T cells in HAAA patients was lower than that of non-HASAA patients (***P<0.05). (HAAA n = 36, Non-HASAA n =  144) (C) HAAA patients had a smaller proportion of CD4+CD25+ T cells than those with non-HASAA (****P<0.05). (The Data was collected since 2003, HAAA n = 28, Non-HASAA n =  112).(D) The absolute number of CD4^+^ T cells in HAAA patients was lower than that of non-HASAA patients (#P<0.05). (E) The absolute number of CD8^+^ T cells in HAAA patients was higher than that of non-HASAA patients (##P<0.05). (F) The proportion of CD4^+^CD25^+^ T cells was lower than that of non-HASAA patients (###P<0.05).

The absolute number of CD4^+^ T cells in HAAA patients ((median, 287±179)×10^6^/L) was lower than that of non-HASAA patients ((median, 475±283)×10^6^/L) (P<0.05). The absolute number of CD8^+^ T cells in HAAA patients ((median, 661±265)×10^6^/L) was higher than that of non-HASAA patients ((median, 414±l89) ×10^6^/L) (P<0.05). The proportion of CD4^+^CD25^+^ T cells ((median, 23.8±11.2)×10^6^/L) was lower than that of non-HASAA patients ((median, 58.8±29.3)×10^6^/L) (P<0.05; Fig. 2).

### Cytokine levels in HAAA

**Figure 3:**
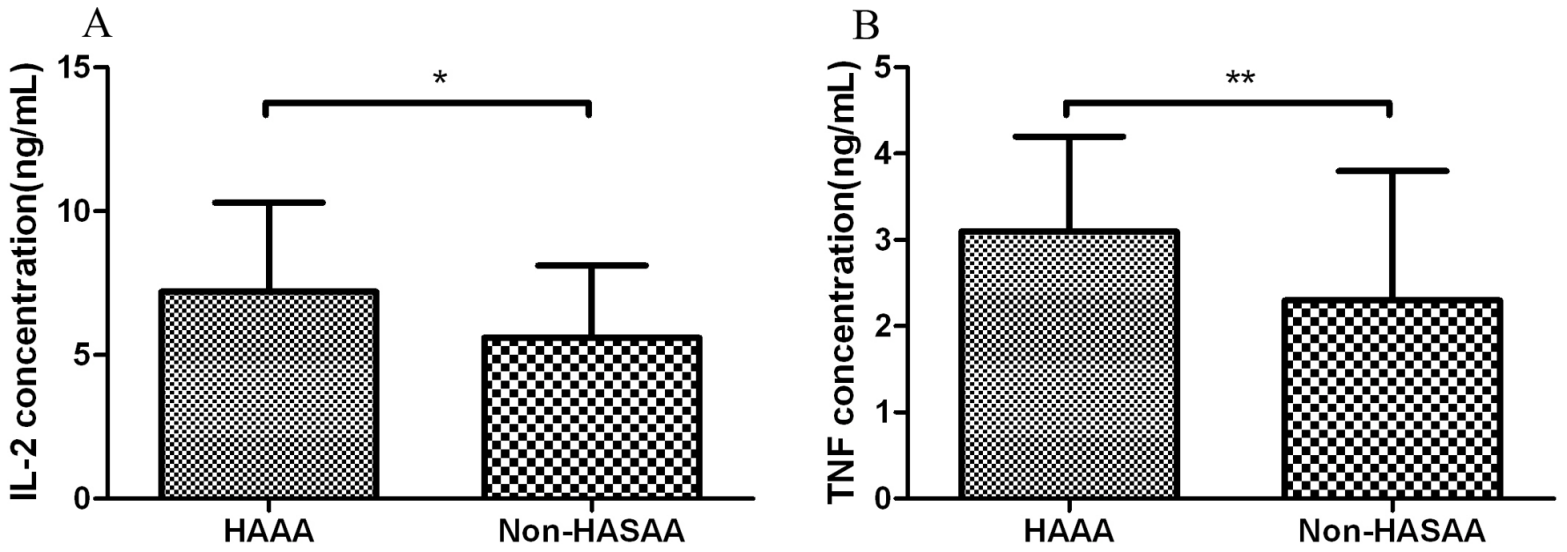
Levels of proflammatory cytokines, IL-2 and TNF-α, in the serum of patients with hepatitis-associated aplastic anemia (HAAA) and non-hepatitis-associated severe aplastic anemia (Non-HASAA). We used a case-control study and collected random cases in non-HASAA patients as contrast group (ratio 1:4). Patients with HAAA had (A) a higher serum IL-2 level (*P<0.05) and (B) a higher serum TNF-α level (**P<0.05) than non-HASAA patients. (HAAA n = 36, Non-HASAA n =  144)

HAAA patients had significantly higher serum IL-2 levels (median, 7.2±3.1 ng/mL) than non-HASAA patients (median, 5.6±2.5 ng/mL; P<0.05). Serum TNF-α levels were also significantly higher in HAAA patients (median, 3.1±1.1 ng/mL) than in non-HASAA patients (2.3±1.5 ng/mL; P<0.05; Fig. 3).

### Infection rate of patients with HAAA

**Figure 4:**
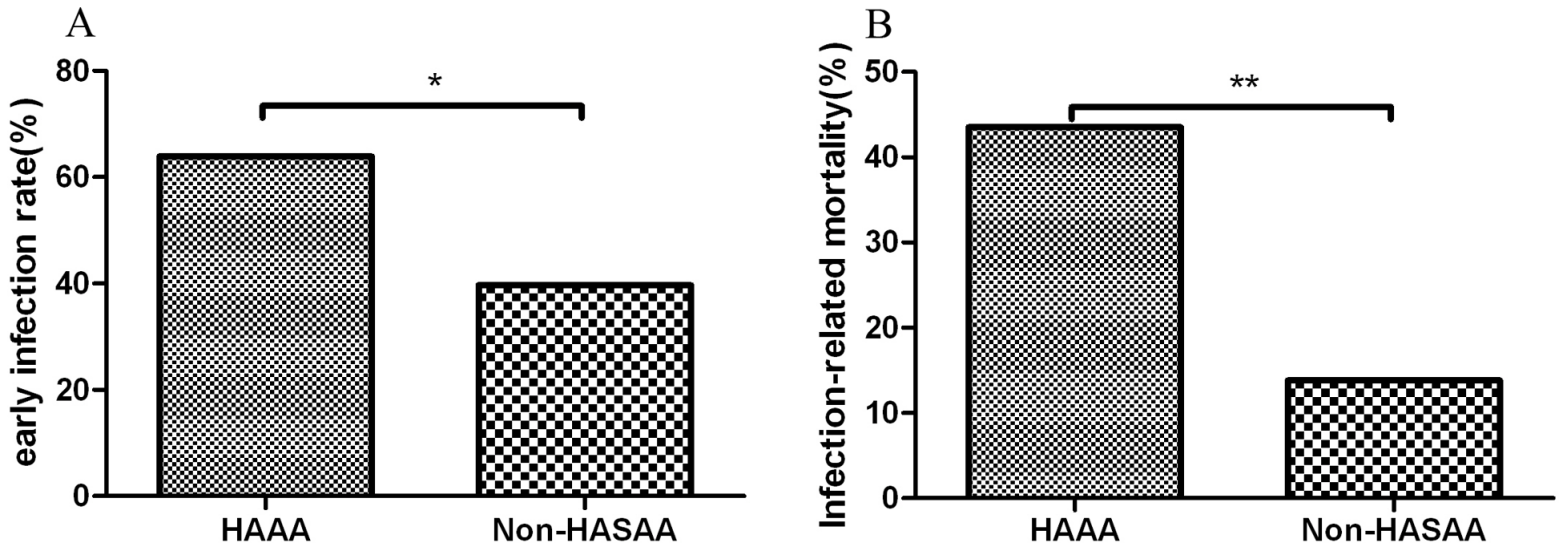
Comparison of early infection rates and infection-related mortality in patients with hepatitis-associated aplastic anemia (HAAA) and non-hepatitis-associated severe aplastic anemia (Non-HASAA). (A) HAAA patients had a higher early infection rate than non-HASAA patients (*P<0.05) and (B) higher infection-related mortality than non-HASAA patients among 2 years (**P<0.05).

Since diagnosis, the infection rate of HAAA patients (63.9%) was higher than that of non-HASAA patients (39.7%) among six months (P<0.05). Most infections in HAAA patients(74%) were polymicrobial, such as more than one bacterial strain, or mixture infections of bacteria and fungi. Among the 23 patients who had infection, 5 were infected by Aspergillus species and predominantly in the lung and sinus(four of them had bacteria infection simultaneously), one was infected by Candida albicans and one was infected by Candida tropicalis. Twenty patients had severe bacterial infections, Klebsiella pneumoniae(5 cases), Escherichia coli(5 cases), Stenotrophomonas maltophilia(3 cases), Pseudomonas aeruginosa(3 cases, Acinetobacter baumannii(2 cases) and methicillin-resistant staphylococcus aureus(2 cases) were the common bacteria. Thirteen patients had bacteremia. In addition to bloodstream infections, lung was the major organ infected. During the 2 years following diagnosis the infection-related mortality was also higher in patients with HAAA (43.5%) than in non-HASAA patients (13.8%; P<0.05; Fig.4).

### Response to therapy and survival rate in patients with HAAA

**Figure 5:**
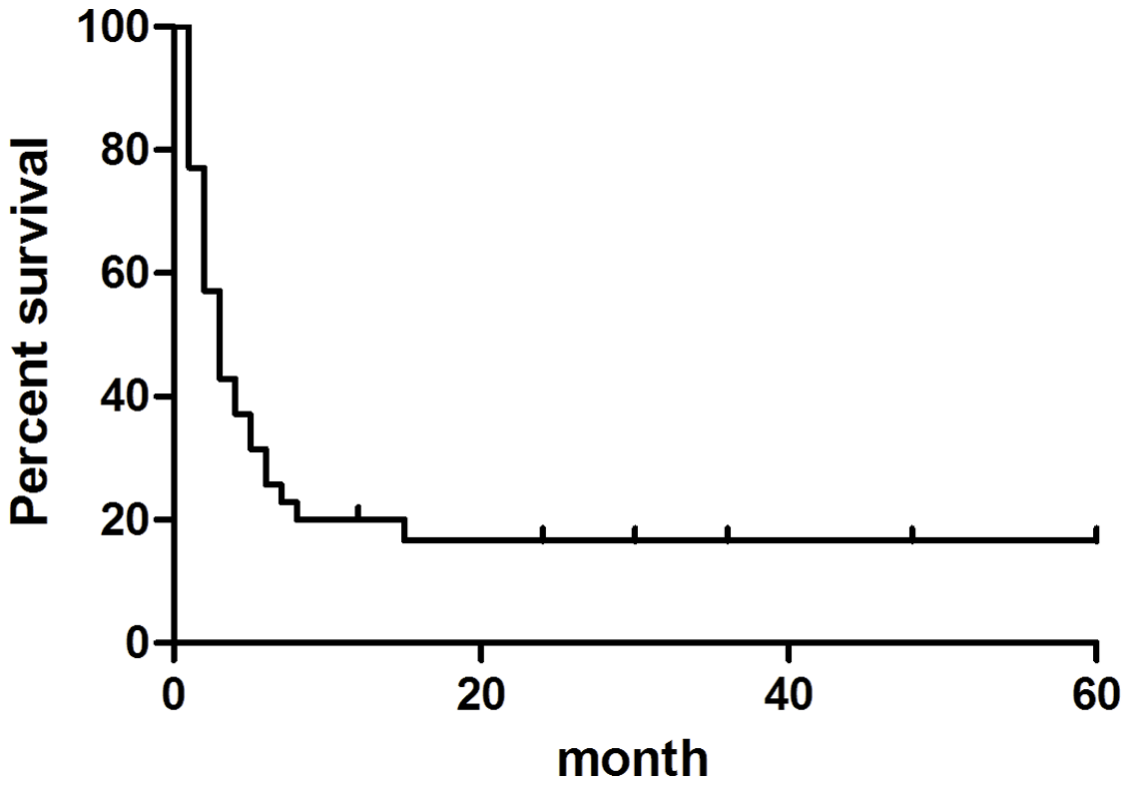
Kaplan-Meier curve showing overall survival of HAAA patients (n = 36). The 2-year survival rate of the HAAA group was 16.6%.

The median follow-up time was 19 months (range, 0.5–60 months). The 2-year survival rate of the HAAA group was 16.6%, and that of the non-HASAA group was 83.2% (Fig. 5). The log-rank test showed that the 2-year survival rate of the HAAA group was significantly lower than that of the non-HASAA group (P<0.01). The results indicate that the HAAA cases are a group with very severe disease and poor prognosis.

## Discussion

HAAA has been reported to constitute 4–10% of SAA cases in the Far East and 2–5% in the West, it might belong to the area of different epidemiology of hepatitis [3]. In a study of 3916 AA patients in Europe between 1990 and 2007, the prevalence of HAAA was 5.4%. Among young patients in this study who received a liver transplantation for fulminant non-A, non-B, non-C hepatitis, 23–28% developed HAAA, whereas of all patients who received a liver transplant, less than 1% went on to develop HAAA [4]. Most studies report that HAAA accounts for 0.1–0.2% of hepatitis cases. In a previous study from China, HAAA accounted for 0.033% of all hepatitis cases, and 3.2% of SAA cases. In the current study the prevalence of HAAA among cases of SAA was 3.8%.

There is currently no consensus on a standard interval between onset of hepatitis and a diagnosis of HAAA and it varies between studies from less than 1 year, to less than 3 months [5] [6] [7]. In the current study, we defined the interval as less than 6 months.

HAAA most often occurs in the recovery period after acute jaundice hepatitis. Safadi *et al.* [8] reported that ALT levels returned to normal at diagnosis of AA in ten out of 17 HAAA patients (59%), and hepatitis resolved partially or completely between 4–12 weeks before AA was noted. In our study, ALT and AST levels decreased in 33 cases (91.7%) prior to the diagnosis of HAAA, including five cases (13.9%) in which the levels returned to normal. The symptoms of hepatitis in these five patients were relatively mild; however, their HAAA symptoms were more severe with aggressive progression and poor prognosis.

As well as hepatitis viruses A, B, C, D, E and G, other viruses such as parvovirus, CMV, Epstein-Barr virus, Transfusion Transmitted virus (TTV) and non-A–E hepatitis virus have also been related to the development of HAAA [3], [9]. Pardi *et al.* [10] showed that HPVB19 infection could lead to hepatic failure and AA. Recently, a parvovirus-like virus, dubbed NIH-CQV, was identified in patients with non-A–E hepatitis by deep sequencing [11]; however, most HAAA cases in this study were seronegative for known hepatitis viruses. Brown *et al*. [1] reported that only one of ten HAAA patients was positive for HBsAb and HBcAb, with the other nine being seronegative for hepatitis A, B and C. Safadi *et al*. [8] found that all 17 HAAA patients studied were seronegative for hepatitis A–E and G, and TTV. They found HPVB19 DNA sequences in two cases, although both serum samples were obtained after blood transfusion, thereby confounding the results. In the current study, two cases of HAAA were caused by an acute hepatitis B virus infection, and there was a history of hepatitis B infection in one case. Hence, we inferred that most HAAA cases were associated with a non-A–E hepatitis virus, or that persistent inflammation after hepatitis altered the immune system even serological detection had turn to negative. A large epidemiological study is now required to reveal the relationship between the etiology of hepatitis and AA.

Clinical features and the response to immunotherapy indicate that immune-mediated factors play a central role in the pathogenesis of HAAA. In a viral infection, the number of activated circulating suppressor T lymphocytes is often increased. Recent studies have demonstrated the expansion of a liver-infiltrating cytotoxic T lymphocyte (CTL) clone in concert with the development of HAAA [12], and that CD8+ Kupffer cells might be important mediators of HAAA [13]. Interferon-γ produced by the T cells and the subsequent cytokine cascades could be involved in HAAA pathogenesis [9]. It indicated that AA and hepatitis had the same mechanism, in which T- cell-mediated immune progress.

In a study of ten cases, Brown *et al.* [1] observed alterations in the T-cell subsets of HAAA patients, including an increase in the proportion of HLA-DR+CD8+ T cells (activated CTL). The ratio of activated CTL decreased after effective treatment, but rose again if IST was discontinued. Young *et al*. [2] investigated the T-cell repertoire (T-cell receptor (TCR) V (beta) chain subfamily) of intrahepatic lymphocytes in HAAA patients before treatment, and found a skewed pattern in the usage of the 21 V (beta) subfamilies in six out of seven samples. The data were similar to those in three of four patients with confirmed viral hepatitis, and higher than those in healthy controls. These results suggested the occurrence of an antigen-driven T-cell expansion in HAAA. With recovery from HAAA, the T-cell repertoire could return to normal. Ikeda *et al.* [14] demonstrated a marked decrease in CD4+ lymphocytes in a patient with HAAA, and in the current study, we found that HAAA patients had a lower ratio of CD4+/CD8+ T cells compared with non-HASAA. Furthermore, we found that in HAAA patients, the serum Th1 cytokines, IL-2 and TNF-α, were at higher levels than in non-HASAA patients.

Compared to healthy controls, the frequency of regulatory T cells (Treg) in AA patients is reduced both in peripheral blood and bone marrow [15]. Defective immunosuppression by Tregs could play a critical role in the pathophysiology of AA. In our study, Tregs could not be exactly distinguished by CD4 and CD25 because of the limited conditions in past decade. But we found that the proportion of CD4^+^CD25^+^ T cells (including Tregs) in the peripheral blood of patients with HAAA was lower than that in patients with non-HASAA. The immune status detection indicated that HAAA patients had more severe T-cell imbalance. The functions of Treg in SAA or HAAA should be studied in the future.

Safadi *et al.* [8] showed that the survival rate of 17 patients with HAAA who received an allogeneic bone marrow transplant (BMT) from a sibling donor was similar to that of 51 non-HASAA patients. Of the ten patients with HAAA who received IST in the study by Brown *et al.* [1], seven had a good response to therapy and the other three died within 1 year, including two from BMT-related complications. In a study of 44 Japanese children with HAAA who received IST, the overall response rate was 70.4% after 6 months and the 10-year overall survival rate was 88.3±4.9% [16]. Hence, combined IST (ATG/ALG) is usually recommended for HAAA patients who lack a suitable donor.

Although there was no significant difference in peripheral reticulocyte count, neutrophil count, bone marrow cellularity and the number of blood transfusions received, between HAAA and non-HASAA patients, our study showed that HAAA patients did not respond as favorably to IST as non-HASAA patients. The 2-year survival rate of HAAA patients was poor, with infection and hemorrhage as the main causes of death.

In summary, in agreement with others we found that HAAA represented a small fraction of SAA cases. Most of the HAAA cases were not associated with any known hepatitis virus. Our findings suggest that T-cell-mediated suppression of bone marrow is more severe in HAAA. Patients with HAAA had worse outcomes than those with non-HASAA. In particular, the survival rate was reduced by severe complications such as infection or hemorrhage.
